# Evaluation of nine formulas for estimating the body surface area of children with hematological malignancies

**DOI:** 10.3389/fped.2022.989049

**Published:** 2022-09-07

**Authors:** Qing Wu, Yan Zhou, Xin Fan, Huan Ma, Wenrui Gu, Fengjun Sun

**Affiliations:** ^1^Department of Pharmacy, Children's Hospital of Chongqing Medical University, National Clinical Research Center for Child Health and Disorders, Ministry of Education Key Laboratory of Child Development and Disorders, Chongqing Key Laboratory of Pediatrics, China International Science and Technology Cooperation Base of Child development and Critical Disorders, Chongqing, China; ^2^Department of Pharmacy, First Affiliated Hospital of Army Medical University, Chongqing, China

**Keywords:** BSA estimation, empirical formulas, young children, hematological malignancies, chemotherapy dose

## Abstract

**Objectives:**

Body surface area (BSA) is an important parameter in clinical practice for children. To find out the most accurate BSA formula for Chinese children, nine formulas were compared.

**Methods:**

This single-center study comprised children who were diagnosed with acute lymphoblastic leukemia and treated with anticancer agents in a specialized children's hospital in China from January 2017 to December 2020. The BSA values were calculated using the formulas from Boyd, Banerjee and Bhattacharya, Costeff, Fujimoto and Watanabe, Haycock, Gehan and George, Mosteller, Stevenson and a Pediatrics textbook. The arithmetic mean of formulas was calculated as the “gold standard” for comparison.

**Results:**

A total of 666 children (389 males and 277 females) were included. All nine formulas showed a strong positive correlation with the “gold standard.” Underestimation was observed with the Banerjee and Bhattacharya, Fujimoto and Watanabe formulas. The Gehan and George formula showed overestimation. Values estimated from the Haycock and Mosteller formulas were the closest to the mean BSA.

**Conclusion:**

The Haycock and Mosteller formulas are the most recommended formulas for Chinese children with hematological malignancies.

## Introduction

Acute lymphoblastic leukemia (ALL) is the most common childhood cancer. Methotrexate (MTX) is the key component of chemotherapy for ALL, and nearly all international treatment regimens incorporate high-dose MTX at ≥1 gram/m^2^ of body surface area (BSA) to obtain adequate concentrations. Most anticancer agents, including MTX, are characterized by a narrow therapeutic index and high interindividual pharmacokinetic variability (1). Drug dosing and dose adjustment are better based on estimates of BSA than actual weight (2, 3). A considerable number of studies describing the absorption, distribution, metabolism and excretion of high-dose MTX relied on BSA (4). Therefore, the determination of BSA is a necessary step in making decisions for critical treatment plans.

Direct measurements of BSA, such as surface integration and three-dimensional scanning, have shown high reliability and repeatability (5). However, this technique is impractical in everyday clinical consultations and emergency practices due to its cumbersomeness, high cost and time consumption. As an alternative, the use of indirect measurements through mathematical formulas that utilize weight and height has been developed as it is easy, fast and inexpensive to use.

A number of BSA formulas have been developed over the past century to simplify its estimation (6–10), but few of them were designed for particular groups, such as children or Asian patients (7, 8). BSA changes significantly with age, as it increases from 0.2 m^2^ at birth to 1.73 m^2^ in adulthood, along with the maturation of organ function (5). An inaccurate BSA may be obtained when these formulas are used to estimate a child's BSA in consideration of differences in body proportions between children and adults. It has been suggested that approximately 30% of patients undergoing chemotherapy will be under dosed and 10% of patients will be overdosed due to inaccurate calculations of BSA, which may cause a reduced cure or other unexpected outcomes (11). Ethnic differences in children's patterns of growth, as to their height and weight, exist. Thus, it is necessary to have evidence for the formula that is most suitable for use and to be able to determine the normal BSA for age and sex within acceptable limits.

The aim of this study was to compare the BSA estimates obtained using 9 formulas for children with ALL undergoing HD-MTX chemotherapy treatment and to determine which of these formulas is most compatible with Chinese children according to age and sex.

## Methods

### Study population

A single-center study that comprised children diagnosed with acute lymphoblastic leukemia treated with HD-MTX in a major tertiary pediatric hospital in China, from January 2017 to December 2020 was carried out. Children <1 year old were excluded because it is challenging to obtain a reliable measure of height, which is prone to measurement errors. The study was approved and registered by the Ethics Committee of this Children's Hospital.

### Data collection

The weights and heights of all the children were measured before the first chemotherapy treatment as part of the preadmission examination. Each child had their measurements taken twice by two different assistants, and the averages were calculated and recorded as the final measurements. Height was recorded to the nearest 0.1 cm, and weight was measured using an electronic scale and recorded to the nearest 0.01 kg.

### BSA formula selection

Nine formulas were compared in the study as shown in Table 1 (6–10, 12–16). These formulas were chosen for the following reasons: enough subjects were used in their formulation, children were included as subjects in the formulation, and either height or weight was included in the formula. It has been proven that weight is the most significant regressor in predicting BSA, yet the inclusion of both weight and height was found to be more explanatory for estimating BSA (17).

**Table 1:** The empirical BSA formulas used in this study.

**No**.	**Formula^#^**	**Equation**
1	Boyd^†^	BSA (cm^2^) = 4.688 × weight (g) ^[0.8168−0.0154 × log(weight)^^(g)]^
2	Banerjee and Bhattacharya	BSA (cm^2^) = 70 × weight (kg) ^0.425^ × height (cm) ^0.725^
3	Costeff (7)	BSA (m^2^) = [4 × weight (kg)] +7)/[90 + weight (kg)]
4	Fujimoto and Watanabe	BSA (cm^2^) = 381.89 × weight (kg) ^0.425^ × height (cm) ^0.362^ (1–5 years); BSA (cm^2^) = 88.83 × weight (kg) ^0.444^ × height (cm) ^0.663^ (over 6 years to old ages)
5	Haycock	BSA (m^2^) = 0.024265 × weight (kg) ^0.5378^ × height (cm) ^0.3964^
6	Gehan and George	BSA (m^2^) = 0.02350 × weight (kg) ^0.51456^ × height (cm) ^0.42246^
7	Mosteller	BSA (m^2^) = $\sqrt{\text{weight }\left(\text{kg}\right)\;\times \text{ height }\left(\text{cm}\right)\;/3600}$
8	Stevenson	BSA (m^2^) = 0.0061 × height (cm) + 0.0128 × weight (kg)−0.1529
9	Pediatrics textbook	BSA (m^2^) = weight (kg) × 0.035 + 0.1 (weight ≤30 kg) BSA (m^2^) = [weight (kg)−30] ×0.02 + 1.05 (weight>30 kg)

# All the formulas listed in Table 1 were demonstrated in original form. In the calculation process, various units had been converted and the unit of BSA was unified to square meter (m^2^).

† The simplified Boyd formula was used in this study, which is based on weight without height.

### Data analysis

The children were classified according to sex and age (in years). The mean values of BSA and their standard deviations (SDs), as well as their 95% confidence intervals, were calculated by substituting weight and height into each of the 9 formulas for the different age and sex categories. As there is no gold standard for BSA against which one can determine the accuracy of any formula, the arithmetic mean of BSA for nine formulas was calculated and designated as the “gold standard.” This method was also described in several previous studies (18).

To determine the significant effect and interaction effects of factors such as age, sex, formulas for BSA predictions and their accuracy, analysis of variance (ANOVA) was conducted at a 0.05 level of significance.

The correlations of the estimated BSA values were determined using regression analyses. The estimated BSA values were compared with the mean BSA value. The accuracy of the estimated BSA values for each of the nine formulas was evaluated using the root mean square error (RMSE), as it combines an assessment of both bias and the spread of data. A lower root mean square error indicated better concordance with the “gold standard” (19).

The relationships between the magnitude and degree of variation in the estimated BSA and mean BSA values were examined by Bland and Altman plots (18). Horizontal lines were drawn at the mean difference (blue solid line) and degree agreements called “95% limits of agreement” (red dotted lines). These dotted lines represent the limits at which the measurement error will be within 95% confidence.

All statistical analyses were performed using IBM SPSS 25.0 for Windows, and a *P* < 0.05 was considered statistically significant.

## Results

### Demographics and anthropometry of the participants

The total sample was narrowed from the raw data of 682 to 666 participants (97.65%) based on the completeness of information entry, such as age, sex, weight, and height. A total of 389 males and 277 females, with an age range of 1–14 years, were enrolled in the study. The children's mean weight was 22.75 ± 11.64 kg, their mean height was 113.66 ± 23.99 cm, and their mean age was 6.29 ± 3.70 years (Table 2).

**Table 2:** Number of subjects, height, weight and mean BSA values.

	**Number of subjects**			**Height (cm)**		**Weight (kg)**		**BSA (m** ^**2**^ **)**	
**Age (years)**				**Males**	**Females**	**Males**	**Females**	**Males**	**Females**
	**Total**	**Males**	**Females**	**Mean ± SD**	**Mean ± SD**	**Mean ± SD**	**Mean ± SD**	**Mean ± SD**	**Mean ± SD**
1	49	29	20	78.53 ± 6.69	79.05 ± 3.12	10.88 ± 1.59	10.13 ± 1.40	0.49 ± 0.05	0.47 ± 0.04
2	89	50	39	89.54 ± 5.83	88.58 ± 4.64	13.34 ± 1.75	12.86 ± 1.84	0.58 ± 0.05	0.56 ± 0.05
3	99	58	41	97.24 ± 5.85	96.18 ± 5.52	15.61 ± 1.99	14.93 ± 2.25	0.65 ± 0.06	0.63 ± 0.06
4	75	39	36	105.03 ± 4.62	104.04 ± 4.57	17.47 ± 2.08	17.23 ± 1.86	0.71 ± 0.06	0.70 ± 0.05
5	58	37	21	112.03 ± 7.70	111.29 ± 13.80	20.51 ± 4.64	19.66 ± 4.69	0.79 ± 0.11	0.77 ± 0.13
6	51	23	28	118.23 ± 6.63	115.81 ± 6.77	22.81 ± 4.74	21.10 ± 3.76	0.86 ± 0.10	0.82 ± 0.10
7	48	32	16	122.61 ± 7.88	119.76 ± 5.58	25.10 ± 5.29	22.68 ± 2.87	0.92 ± 0.11	0.86 ± 0.07
8	40	23	17	129.18 ± 7.74	127.28 ± 4.90	28.34 ± 7.86	26.18 ± 3.47	1.00 ± 0.16	0.96 ± 0.08
9	29	20	9	131.29 ± 12.63	135.72 ± 9.39	28.82 ± 7.77	29.70 ± 4.67	1.01 ± 0.17	1.05 ± 0.10
10	30	19	11	141.76 ± 6.30	138.00 ± 8.04	37.20 ± 10.49	32.27 ± 6.89	1.19 ± 0.19	1.10 ± 0.14
11	17	11	6	142.35 ± 7.02	139.94 ± 6.76	34.25 ± 7.17	31.79 ± 3.16	1.14 ± 0.14	1.10 ± 0.07
12	38	22	16	152.49 ± 9.60	150.81 ± 6.69	41.78 ± 10.80	41.29 ± 5.78	1.30 ± 0.20	1.29 ± 0.11
13	21	10	11	161.93 ± 6.76	152.39 ± 12.03	52.41 ± 12.38	42.06 ± 8.77	1.50 ± 0.21	1.31 ± 0.17
14	22	16	6	161.47 ± 11.18	159.19 ± 1.31	47.66 ± 9.43	49.47 ± 5.77	1.43 ± 0.18	1.44 ± 0.08
Total	666	389	277	114.70 ± 125.10	112.12 ± 23.90	23.57 ± 12.53	21.76 ± 10.77	0.85 ± 0.30	0.81 ± 0.27

Table 2 also shows the overall mean BSA values according to sex and age. The mean estimated BSA showed an increasing trend with aging. It was also observed that from age 1–14, males always had higher BSA values than females, except at age 9 and age 14.

### Main effects and interaction effects of the BSA values

ANOVA was conducted to test the main effects and interaction effects of BSA, including age, sex, and model factors. Table 3 shows the ANOVA results, and the main effects of age, sex and model factors were statistically significant (*P* < 0.001). The two-way interaction effect (age-sex) was also statistically significant (*P* < 0.001). Other two-way interaction effects (sex-model, age-model) and a three-way interaction effect (age-sex-model) were found to be not significant (*P* > 0.05)

**Table 3:** Analysis of variance results for age, sex and formula main effects and interaction effects.

**Variable**	**SS**	**df**	**MS**	***F***	***P*-value**
Age	396.402	13	30.492	2598.456	<0.001*
Sex	1.412	1	1.412	120.353	<0.001*
BSA formula	1.468	8	0.184	15.638	<0.001*
Age × sex	2.089	13	0.161	13.697	<0.001*
Age × formula	0.905	104	0.009	0.742	0.977
Sex × formula	0.002	8	0	0.02	1.000
Age × sex × formula	0.084	104	0.001	0.069	1.000
Error	67.381	5,742	0.013		
Total	495.012	5,993			

SS, sum of squares; df, degrees of freedom; MS, mean squares; F, F ratio; BSA, body surface area.

* *P* < 0.05.

### Correlation between different BSA formulas

The correlation coefficient, *R*^2^ and RMSE that were calculated using regression analyses are shown in Table 4. Subgroup analyses by sex are also displayed in Table 4. All BSA formulas showed great correlation with “the gold standard,” with an *R*^2^ range from 0.981 to 1.000 (*P* < 0.001 for each). The Haycock formula had the lowest RMSE of all nine formulas in the male, female and all subject groups, which indicated that it was closest to the predicted BSA value. The Mosteller formula was just behind it, with an *R*^2^ of 0.999 and an RMSE of 0.012. In contrast, the pediatrics textbook formula and Fujimoto and Watanabe formulas had the highest RMSE.

**Table 4:** Correlation between the BSA values of the different formulas and the mean value.

**Formula**	**All subjects (*****N =*** **666)**				**Males (*****n =*** **389)**				**Females (*****n =*** **277)**			
	**Correlation**	***R^2^***	**RMSE**	***P* value**	**Correlation**	***R* ^2^**	**RMSE**	***P* value**	**Correlation**	***R^2^***	**RMSE**	***P* value**
	**(r)**				**(r)**				**(r)**			
Boyd	0.997	0.994	0.024	<0.001	0.997	0.994	0.025	<0.001	0.997	0.994	0.023	<0.001
Banerjee and Bhattacharya	0.998	0.996	0.027	<0.001	0.998	0.996	0.027	<0.001	0.998	0.996	0.026	<0.001
Costeff	0.998	0.996	0.021	<0.001	0.998	0.996	0.021	<0.001	0.998	0.995	0.020	<0.001
Fujimoto and Watanabe	0.990	0.981	0.057	<0.001	0.990	0.981	0.058	<0.001	0.990	0.981	0.054	<0.001
Haycock	1.000	1.000	0.009	<0.001	1.000	1.000	0.010	<0.001	1.000	0.999	0.009	<0.001
Gehan and George	1.000	1.000	0.023	<0.001	1.000	1.000	0.023	<0.001	1.000	0.999	0.023	<0.001
Mosteller	1.000	0.999	0.012	<0.001	1.000	0.999	0.012	<0.001	1.000	0.999	0.011	<0.001
Stevenson	0.997	0.994	0.022	<0.001	0.997	0.995	0.022	<0.001	0.997	0.993	0.022	<0.001
Pediatrics textbook	0.994	0.983	0.032	<0.001	0.995	0.990	0.032	<0.001	0.993	0.987	0.032	<0.001

r, correlation coefficient; RMSE, root mean squared error; the lower the RMSE value is, the better the accuracy of the formula to predict the gold standard.

### Agreement between different BSA formulas

**Figure 1:**
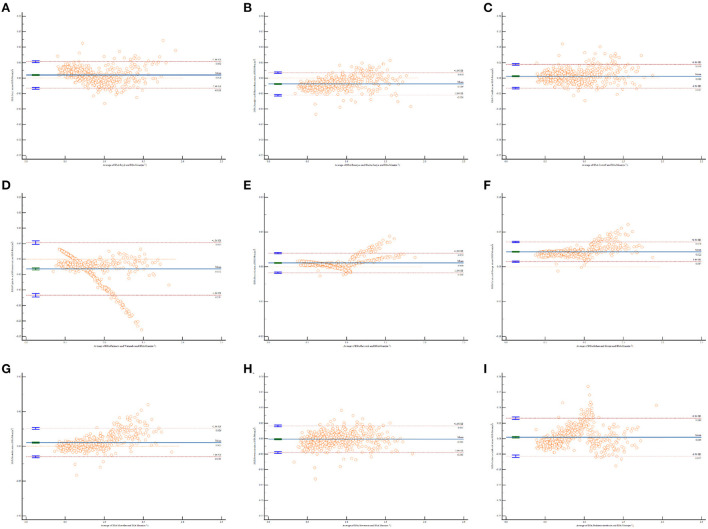
Bland and Altman plots of the differences in body surface area (BSA) values calculated by different formulas against the mean BSA values. The horizontal x-axis shows the average of BSA calculated by each formula and mean BSA, AND THE vertical y-axis shows the D-value of BSA calculated by each formula and mean BSA. **(A)** BSA calculated by Boyd formula vs. the mean BSA values; **(B)** BSA calculated by Banerjee and Bhattacharya formula vs. the mean BSA values; **(C)** BSA calculated by Costeff formula vs. the mean BSA values; **(D)** BSA calculated by Fujimoto and Watanabe formula vs. the mean BSA values; **(E)** BSA calculated by Haycock formula vs. the mean BSA values; **(F)** BSA calculated by Gehan and George formula vs. the mean BSA values; **(G)** BSA calculated by Mosteller formula vs. the mean BSA values; **(H)** BSA calculated by Stevenson formula vs. the mean BSA values; **(I)** BSA calculated by Pediatrics textbook formula vs. the mean BSA values.

The Bland and Altman plots for all comparisons are shown in Figure 1. The solid line in the center indicates the mean bias, and the dashed outer lines indicate the 95% limit of agreement. It is clear that the limits of agreement for eight formulas included the mean BSA value, which indicates an agreement with BSA estimation to some degree, except for the Gehan and George formula. The Bland–Altman graph for the Haycock (Figure 1E) and Mosteller (Figure 1G) formulas produced the two smallest biases and were consistent with the mean BSA from low to high values. The Fujimoto and Watanabe formula was the only formula to show an obvious systematic error. According to the Bland–Altman graph, the Fujimoto and Watanabe formula has a tendency to underestimate BSA in older children. The Bland–Altman graph for the Mosteller, Haycock, and Gehan and George formulas (Figures 1E–G) shows a relatively narrower line of 95% limits of agreement compared with the other formulas.

## Discussion

Body surface area was developed as a metric to use in the modulation of various pharmacological therapies and a standard tool by which to index various physiologic measurements (20, 21). In the field of oncology, the dosage of anti-neoplastic drugs is generally based on estimated BSA (22). Recently, chemotherapy dose calculations according to BSA were criticized, and some other body-size measures have been proposed, such as lean body mass, ideal body weight, adjusted ideal body weight and body mass index. Some measures that might be used in conjunction with or instead of BSA have already been explored for some agents, such as PK monitoring and enzyme phenotyping (23, 24). However, until such dosing techniques are developed and standardized, we have to depend on BSA-based dose calculation for most chemotherapy agents, so long as it still has the advantage of concision and preciseness.

Correct estimation of children's BSA is a necessary step in pediatric practice, particularly in the dosing of anticancer agents (3, 25). The action of the drugs and their pharmacokinetics in children, especially young children, can be very different from those in adults (26). Therefore, the accurate dosing of medicine for children under 18 years old is preferably made using BSA. A number of formulas are available for estimation, but they do not provide standard calculation and restriction details, such as ethnicity, age, sex and other factors. Another problem is that most BSA formulas were based on the general population of healthy children. However, BSA estimation is most frequently used for sick children with malignant tumors or congenital heart disease. These children are malnourished and often excluded in large studies.

The aim of this study was to validate existing formulas for estimating BSA values in children undergoing chemotherapy. Moreover, we hope the study can provide overall BSA estimates for these patients. In all, nine formulas were compared according to age and sex. The study showed that the BSA values from all nine formulas had a great correlation with the mean BSA value (as the gold standard), and the BSA values increased as age increased. The Haycock, Mosteller and Gehan and George formulas had the highest correlation coefficient (*R*^2^ = 0.999), and the former was considered the most accurate because it had the lowest root mean square error of all nine formulas.

According to the Bland and Altman plots, the limits of agreement for the Gehan and George formula did not include the mean BSA value, which indicated a considerable disagreement in its estimation, although it produced a fairly small bias. This formula seemed to overestimate BSA when compared to the mean BSA value. One possible reason might be that Gehan and George carried out their study based on healthy children, while our study involved young children with hematological malignancies (14). Children with chemotherapy often experience liver and kidney failure due to toxicity of anticancer agents. This can, in turn, influence their metabolic capacity for drugs. An overestimation of BSA may lead to overdosing, which results in more side effects and organ damage.

The Haycock and Mosteller formulas produced the first and third smallest biases in the Bland and Altman plots. It was found that the Haycock and Mosteller formulas performed best for our study subjects. This conclusion was supported by Orimadegun and Omisanjo (27), who found that the Mosteller and Boyd formulas provide the most accurate BSA values for Nigerian children.

The Mosteller formula has been studied by several papers since it was established in 1987 (3, 27, 28). The greatest competitive advantage of the Mosteller formula is its conciseness. It is easy to remember and use with the help of a calculator with a square root function. Lam and Leung (29) calculated the BSA of 168 children aged from 1 month to 14 years using the Mosteller formula and confirmed that it was equally applicable to children.

The precise prediction of the Haycock formula can be attributed to the sound formulation of the equation with newborns, infants, children and adults (15). The study included 81 individuals of widely varying physiques, ranging from very thin to obese, and Black, Hispanic and White children were included. The surface area was calculated by means of the geometric method, and the validity of the formula was tested in three ways. By the above modeling methods, the accuracy and flexibility of the Haycock formula is guaranteed.

The Fujimoto and Watanabe and Banerjee and Bhattacharya formulas both showed underestimation in the Bland and Altman plots (bias<0). The underestimation of BSA can lead to serious consequences. For example, underdosing chemotherapeutic agents due to the inaccurate calculation of BSA may cause a reduced cure or other unexpected outcomes (11). It is believed that the limited number of subjects (*n* = 13, including 11 males and 2 females) caused the inaccuracy of the Banerjee and Bhattacharya formula (13). The Fujimoto and Watanabe formula was first introduced over 50 years ago (8), which is perhaps why it is no longer suitable for the current Asian population.

Three formulas that only use weight were included. These were the simplified Boyd formula, Costeff formula and Pediatrics textbook formula. The original Boyd formula was developed from a large sample of 1,114 individuals, including 401 children, in 1935 (6) and later simplified as weight is the only determining factor (16). Of these three formulas, the Costeff formula was in agreement with the mean BSA value in the Bland and Altman plots, while the other two showed a relatively wider line of 95% limits of agreement. Therefore, it seemed that BSA formulas using weight and height can be more accurate. The pediatrics textbook formula was from “Zhu Futang Practice of Pediatrics,” which is an authorized textbook for pediatrics in China. As the pediatrics textbook formula is very simple, it is widely used in BSA calculations clinically.

The Stevenson formula is another widely used formula in China. It was established in 1937 by Paul H. Stevenson, and is a modification of the Du Bois formula with data from the Chinese population (12). This formula has been in use for decades, but few studies have validated its accuracy. Our study confirmed that it has a great correlation with the mean BSA but has a wider line of 95% limits of agreement. The somatotype of Chinese individuals has obviously changed along with significant improvements in nutrition status over the past several decades. These changes make the Stevenson formula less practical at the present time.

One important limitation of this study is that there were no direct BSA measurements, such as those using a three-dimensional scanner. To compensate for this disadvantage, the mean BSA value based on nine formulas were utilized as the gold standard. This approach has been validated in many previous studies (18, 19). The second limitation is that we did not explore the effect of body mass on BSA prediction. Considering that the population of the study was children with ALL, the proportion of overweight children was too small to make valid comparisons among normal, underweight and obese populations in the same age group.

## Conclusion

In summary, this study evaluated nine BSA formulas for children. Although all of the predictions showed positive correlations with the mean BSA value, the Haycock formula estimated the closest values to the mean BSA value. Therefore, the use of the Haycock formula is recommended for estimating body surface area among Chinese children with ALL. However, further study is needed to explore the effect of body mass on BSA.

## Data availability statement

The original contributions presented in the study are included in the article/Supplementary material, further inquiries can be directed to the corresponding author/s.

## Ethics statement

The studies involving human participants were reviewed and approved by the Ethics Committee of Children's Hospital of Chongqing Medical University (No. 2021-69). Written informed consent to participate in this study was provided by the participants' legal guardian/next of kin.

## Author contributions

QW contributed to conceptualization, methodology, software, statistical analysis, and writing of original draft preparation. YZ and XF contributed to methodology, acquisition of data, review, and editing. HM contributed to software, data curation, and statistical analysis. FS and WG contributed to conceptualization, review and editing, supervision, and project administration. All authors have read and agreed to the published version of the manuscript.

## Conflict of interest

The authors declare that the research was conducted in the absence of any commercial or financial relationships that could be construed as a potential conflict of interest.

## Publisher's note

All claims expressed in this article are solely those of the authors and do not necessarily represent those of their affiliated organizations, or those of the publisher, the editors and the reviewers. Any product that may be evaluated in this article, or claim that may be made by its manufacturer, is not guaranteed or endorsed by the publisher.
